# Phytochrome A signal transduction 1 and CONSTANS‐LIKE 13 coordinately orchestrate shoot branching and flowering in leafy *Brassica juncea*

**DOI:** 10.1111/pbi.13057

**Published:** 2019-02-20

**Authors:** Sidra Tul Muntha, Lili Zhang, Yufeng Zhou, Xuan Zhao, Zhongyuan Hu, Jinghua Yang, Mingfang Zhang

**Affiliations:** ^1^ Laboratory of Germplasm Innovation and Molecular Breeding Institute of Vegetable Sciences Zhejiang University Hangzhou China; ^2^ Key Laboratory of Horticultural Plant Growth, Development & Quality Improvement Ministry of Agriculture Hangzhou China; ^3^ Zhejiang Provincial Key Laboratory of Horticultural Plant Integrative Biology Hangzhou China

**Keywords:** *Brassica juncea*, branching, phytochrome A signal transduction 1, CONSTANS‐LIKE 13, flowering

## Abstract

Branching is a major determinant of crop yield, and enables vigorous shoot growth and the production of a dense canopy. Phytochrome A signal transduction 1 (PAT1) positively regulates phytochrome A signal transduction in response to light, but its effects on branching remain unknown. In this study, we mapped *PAT1*, and revealed a previously unknown role related to branching and flowering in leafy *Brassica juncea*. Earlier and increased branching was observed when *PAT1* expression was down‐regulated, implying that PAT1 negatively regulates shoot branching. Additionally, down‐regulated *PAT1* expression reversed the inhibited branching induced by far‐red light, suggesting PAT1 is involved in the shade avoidance response. PAT1 negatively regulated branching only after bud initiation. The observed interaction between PAT1 and BRC1 implied that PAT1 influences bud outgrowth in a BRC1‐dependent manner. Biochemical and genetic evidence indicate that PAT1 directly interacts with CONSTANS‐LIKE 13 (COL13), which negatively regulates flowering, with the resulting PAT1–COL13 complex mediating shoot branching and flowering. Our findings reveal a new crosstalk modality between phytochrome signalling and flowering pathways during the regulation of shoot branching and flowering. The data presented herein may be useful for future studies involving the editing of the GRAS family transcription factor *PAT1* gene to enhance crop productivity and enable earlier harvesting.

## Introduction

Shoot branching, like tillering in grasses, significantly influences plant architecture, which is an important factor affecting crop yield (Janssen *et al*., 2014; Kebrom *et al*., 2013; Wang *et al*., 2018). During shoot branching, axillary meristems are formed in the axil of each leaf, with the potential to develop into a branch. Axillary bud activity has long been an essential consideration for crop breeders (Springer, 2010) because it fundamentally impacts yield by influencing the number of branches and inflorescences as well as inflorescence development. Branching is controlled by complex interactions among phytohormonal, developmental, and environmental factors (Janssen *et al*., 2014; Kebrom *et al*., 2013). Axillary bud growth is regulated by long‐distance signalling, which is mediated mainly by auxin, cytokinin, and strigolactone (Domagalska and Leyser, 2011). Auxin, which is one of the most extensively studied phytohormones, is synthesized in the shoot apex and controls bud dormancy. It mediates strigolactone levels by increasing *CCD7* and *CCD8* transcription, and inhibits cytokinin biosynthesis by suppressing the expression of the cytokinin biosynthesis gene *ISOPENTENYL TRANSFERASE* (Brewer *et al*., 2009; Leyser, 2009).

Several factors affect branching in plants, including nutrient availability (i.e., starvation conditions), light quantity and quality, temperature, and (potentially) sugar levels (Janssen *et al*., 2014; Kebrom *et al*., 2013). Light is critical for branching, and plants sense different light‐related stimuli and modulate bud outgrowth and branch development accordingly. Plant responses to the red:far‐red light ratio (R:FR), which is affected by the shade due to neighbouring plants, are collectively part of the shade avoidance syndrome (SAS), and are associated with the phytochrome family of photoreceptors (Krishna Reddy and Finlayson, 2014). A previous study revealed that in response to decreased exposure to light, the SAS is likely to break the dormancy of axillary buds, resulting in elongated stems and early flowering (Ballare, 1999). The role of phytochrome B (PHYB) in sensing the R:FR signal has been studied extensively. Additionally, the link between PHYB and branching has been demonstrated by analysing a *phyB* null mutant and the response to shade, which is signalled by a low R:FR ratio (Smith, 1995). Loss‐of‐function mutations to PHYB in *Arabidopsis thaliana* and *Sorghum bicolor* exhibit decreased bud outgrowth and branching, suggesting that the R:FR ratio mediates bud outgrowth (Finlayson *et al*., 2010; Kebrom *et al*., 2006). However, in rice, a mutation to the gene encoding phytochrome A (*PHYA*) does not affect bud outgrowth (Takano *et al*., 2001), while in pea, a mutated *PHYA* considerably increases branching (Weller *et al*., 1997). In cotton, *PHYA* RNA‐interference lines exhibit increased shoot branching, early flowering, and increased crop yield (Abdurakhmonov *et al*., 2014). Moreover, BRC1/TB1 proteins, which are conserved across eudicots and monocots, integrate the effects of phytohormonal and light signals that control branching (Domagalska and Leyser, 2011; Janssen *et al*., 2014; Kebrom *et al*., 2013).

Phytochrome A signal transduction 1 (PAT1), which belongs to the GRAS [GAI (Gibberellin‐insensitive), RGA (Repressor of ga1‐3), and SCR (Scarecrow)] transcription factor family, contributes to PHYA signal transduction (Bolle *et al*., 2000; Torres‐Galea *et al*., 2013). In *A. thaliana*, the *PAT1* clade includes six genes (*PAT1, SCL1, SCL5, SCL8, SCL13,* and *SCL21*), all of which are involved in the PHYA signalling pathway, with the exception of *SCL13*, which affects PHYB signalling (Torres‐Galea *et al*., 2006). A recent study suggested that the ERF115–PAT1 complex is crucial for plant regeneration, particularly in the recovery and indeterminate growth of the root meristem (Heyman *et al*., 2016). However, it remains unclear whether PAT1 is involved in shoot branching.

*Brassica juncea*, which is an allopolyploid, has multiple agricultural uses, including as a vegetable (leaf), oilseed, and condiment crop (Yang *et al*., 2016, 2018b). Shoot branching is a critical factor affecting the yield of many leafy *B. juncea* varieties. In this study, we mapped a *PAT1* ortholog as a candidate gene associated with shoot branching in leafy *B. juncea*. Down‐regulated *PAT1* expression significantly increased the number of branches on branching (XLH) and non‐branching (T84‐63) leafy *B. juncea* lines. The data presented herein revealed that PAT1 can alleviate the suppressed branching induced by far‐red light, suggesting that it may contribute to the SAS pathway. Furthermore, we confirmed that PAT1 physically interacts with COL13 and negatively regulates branching and flowering.

## Results

### Genetic analysis of branching in *Brassica juncea*

**Figure 1:**
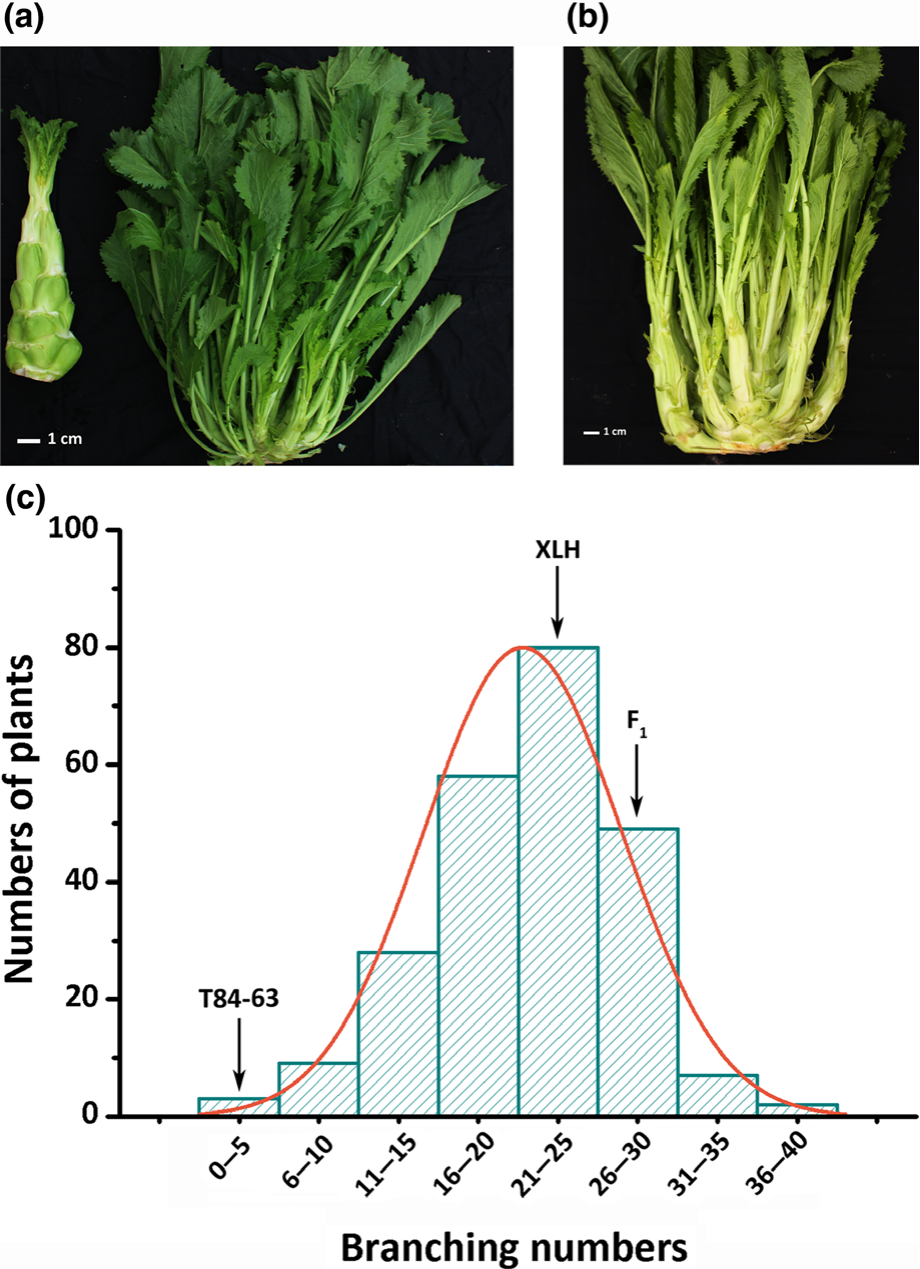
Phenotype and segregation of shoot branching in *Brassica juncea*. (a) Branching line (XLH, right) and non‐branching line (T84‐63, left). (b) Branching phenotype of F_1_ plants from cross between XLH and T84‐63. (c) Distribution of number of branches on 236 F_2_ individuals. Average numbers of branches for XLH (23.3), T84‐63 (0), and XLH × T84‐63 F_1_ hybrid (25.9) are indicated with arrows.

*Brassica juncea* branching (XLH) and non‐branching (T84‐63) lines were crossed to generate segregating populations (Figure 1a,b). On average, the XLH, T84‐63, and F_1_ plants had 23.3, 0, and 25.9 branches, respectively. The fact that the F_1_ plants had the most branches is likely because of heterosis. An analysis of the number of branches among 236 F_2_ plants revealed a largely normal distribution, implying that branching is controlled by a quantitative trait locus (Figure 1c; Table S1).

### Bulked segregant analysis by resequencing identifies PAT1 as candidate branching gene

**Figure 2:**
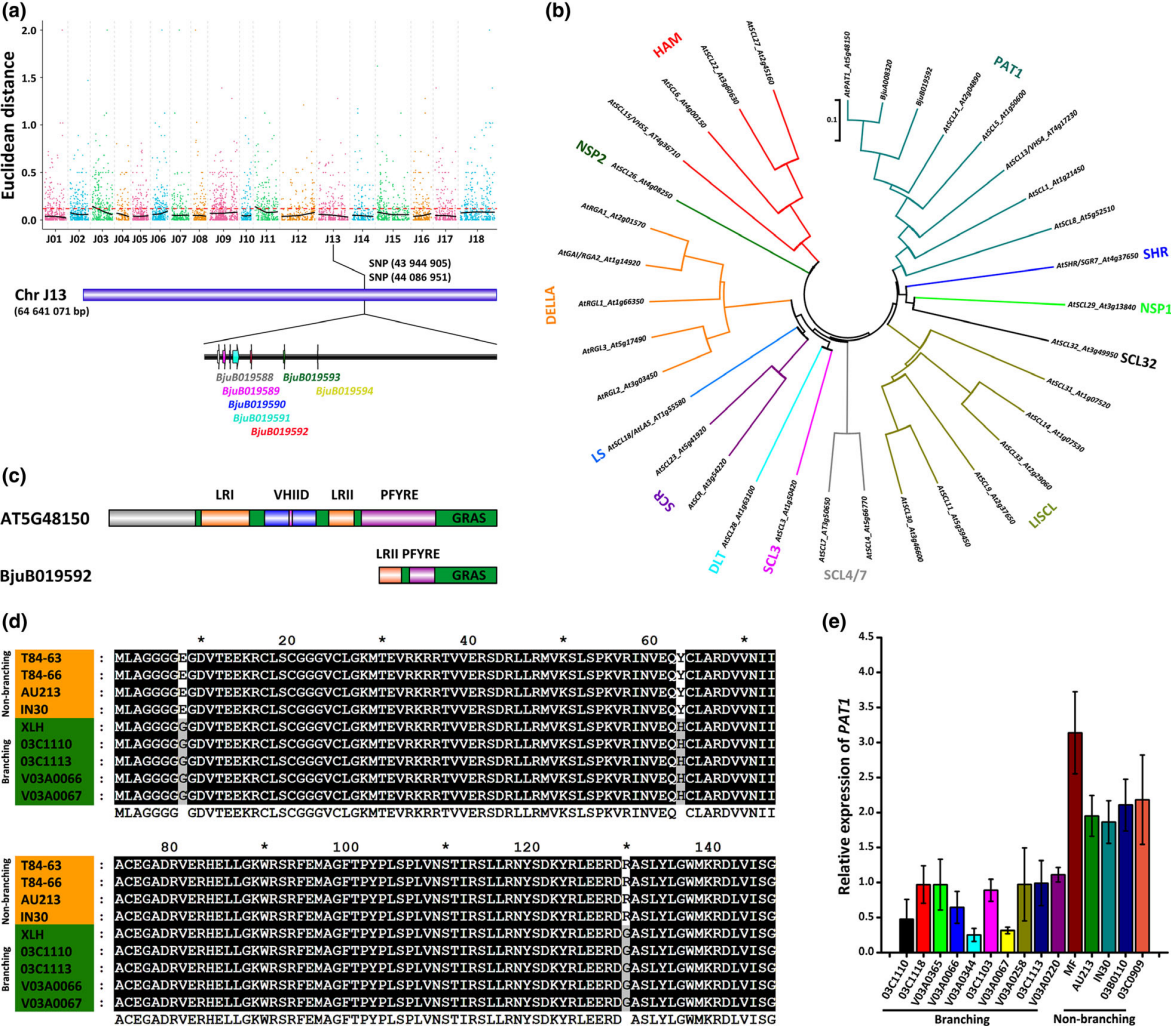
Mapping of *Brassica juncea* shoot branching candidate genes based on bulked segregant analysis. (a) Single nucleotide polymorphism‐based association analysis of branching and non/less‐branching gene pools. (b) Phylogenetic analysis involving BjuB019592 and GRAS transcription factor family genes. GRAS subfamilies are shown. (c) Conserved domains and motifs in BjuB019592 and its *Arabidopsis thaliana* ortholog (At5G48150). (d) Genotyping of non‐synonymous substitutions of BjuB019592 in branching and non‐branching *B. juncea* lines. (e), BjuB019592 expression in branching and non‐branching *B. juncea* lines.

The branching‐associated gene was mapped by resequencing the F_2_ branching and non‐branching bulks resulting from the XLH and T84‐63 cross. High‐throughput sequencing with the Illumina HiSeq 2500 system generated approximately 6.5 and 6.9 million clean reads for the branching and non‐branching pools, respectively (Table S2). We subsequently identified more than 40 000 single nucleotide polymorphisms (SNPs) in each pool based on a reference genome (Figure S1; Tables S3 and S4). An association analysis involving Euclidean Distance revealed two main branching‐related regions (Table S5). After annotating genes and conducting an enrichment analysis of the genes in the associated regions with nr, Swiss‐Prot, Gene Ontology (GO), Cluster of Orthologous Groups (COG), and Kyoto Encyclopedia of Genes and Genomes (KEGG) databases (Figure S2; Table S6), we identified BjuB019592 as a candidate branching‐related gene located between SNPs (43 944 905–44 086 951) on chromosome J13 (Figure 2a; Table S7). Additionally, BjuB019592 encoded 149 amino acids, with 66% homology with an *A. thaliana* PAT1 (At5G48150, 409 amino acids). We constructed a neighbour‐joining phylogenetic tree comprising the 33 *A. thaliana* GRAS transcription factor genes as well as BjuB019592 and its homoeolog, BjuA008320 (Figure 2b). The tree indicated that BjuB019592 is an ortholog of *A. thaliana PAT1*. An analysis of the conserved domains between BjuB019592 and At5G48150 indicated that BjuB019592 includes the GRAS domain as well as the LRII and PFTRE motifs of PAT1 proteins, but lacks the LRI and VHIID motifs (Figure 2c). In the *PAT1* (BjuB019592) coding sequence (CDS), three non‐synonymous SNPs exhibited the expected segregation between the branching (XLH) and non‐branching (T84‐63) lines (Figure S3). An analysis of *PAT1* (BjuB019592) in another four branching and three non‐branching *B. juncea* lines revealed that in all four branching lines (03C1110, 03C1113, V03A0066, and V03A0067), three non‐synonymous SNPs (23, 187, and 388) resulted in Glu, Tyr, and Arg, respectively. Meanwhile, in all three non‐branching lines (84‐66, AU213, and IN30), the corresponding SNPs resulted in Gly, His, and Gly, respectively (Figure 2d; Figure S3). Moreover, *BjPAT1* transcript levels were lower in *B. juncea* branching lines than in non‐branching lines (Figure 2e).

### Functional analysis of PAT1 confirms that it negatively regulates branching

**Figure 3:**
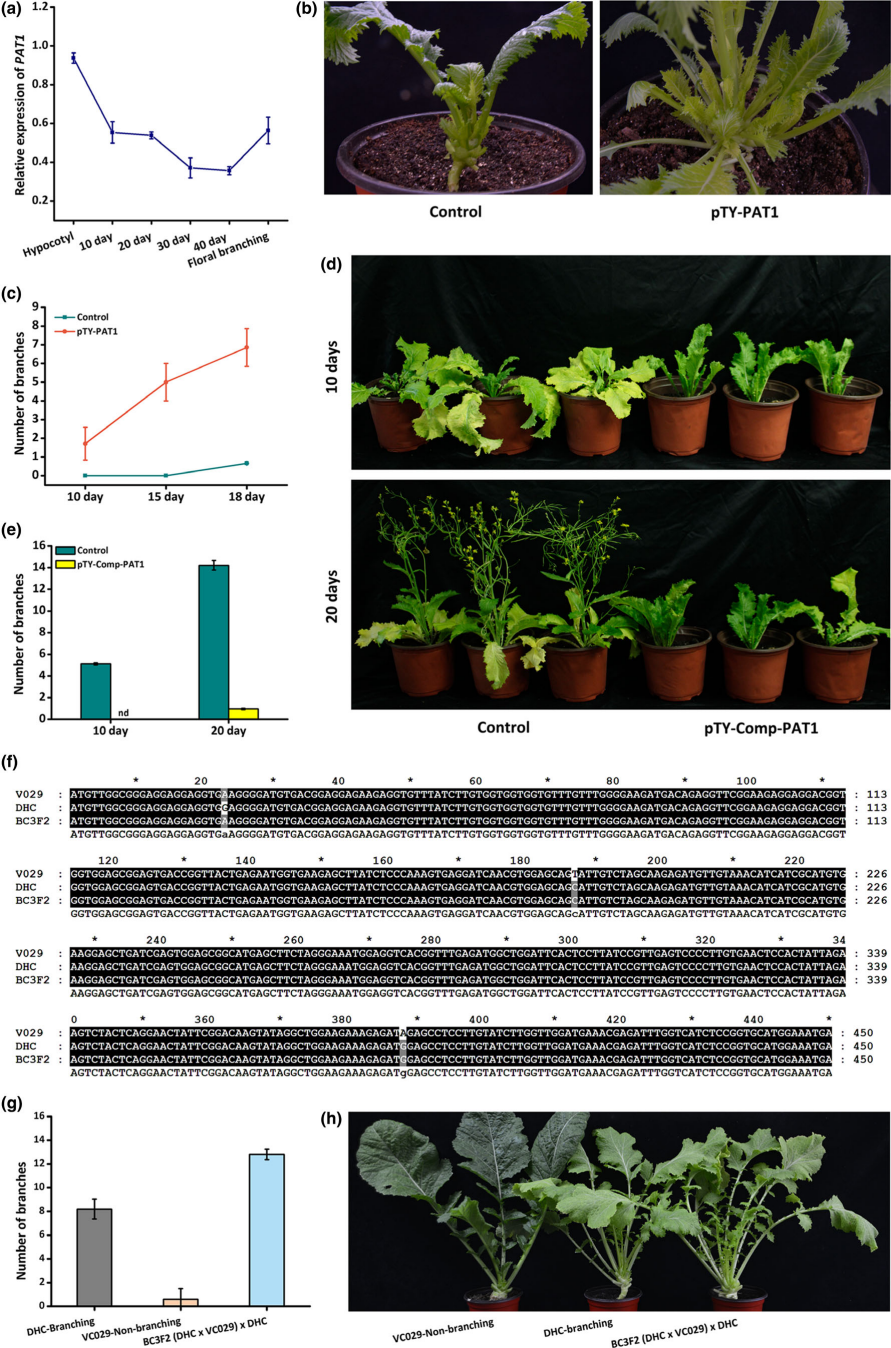
Analysis of *PAT1* functions related to regulation of *Brassica juncea* shoot branching. (a) Developmental expression patterns of *PAT1* in XLH line. (b) Branching phenotypes of pTY‐PAT1 (XLH) plants with down‐regulated *PAT1* expression and control (XLH) plants. (c), Number of branches on pTY‐PAT1 (XLH) plants with down‐regulated *PAT1* expression and control plants during developmental stages. (d) Branching phenotypes of pTY‐Comp‐PAT1 plants with complementary expression of *PAT1* from non‐branching (XLH) plants and control (XLH) plants. (e) Number of branches on pTY‐Comp‐PAT1 plants with complementary expression of *PAT1* from non‐branching (XLH) plants and control (XLH) plants. (f) Genotyping of non‐synonymous substitutions in BjuB019592 in BC_3_F_2_ backcross line resulting from crossing of branching and non‐branching lines and subsequent crossing with a branching line as recurrent parent. (g) Branching phenotypes of BC_3_F_2_ backcross line resulting from crossing of branching and non‐branching lines and subsequent crossing with a branching line as recurrent parent. (h) Number of branches for BC_3_F_2_ backcross line resulting from crossing of branching and non‐branching lines and subsequent crossing with a branching line as recurrent parent.

The observed continuous down‐regulation of *BjPAT1* expression before flowering suggested that it encodes a negative regulator of branching (Figure 3a). We further characterized the regulatory functions of *BjPAT1* affecting shoot branching in leafy *B. juncea* by using turnip yellow mosaic virus (TYMV)‐induced gene silencing (VIGS) to silence *BjPAT1* expression. The associated vector does not affect vegetative growth and flowering in *A. thaliana* and *B. juncea* (Pflieger *et al*., 2008; Shopan *et al*., 2017). In VIGS plants treated with the pTY‐S virus vector designed to silence *PAT1* expression (pTY‐PAT1), *BjPAT1* expression was significantly down‐regulated (Figure S4). Additionally, the expression levels of *BjPAT1* homologs (BjuA008320, BjuB042895, BjuB044594, and BjuB007739) were not significantly altered when BjuB019592 expression was down‐regulated (Figure S5). The XLH plants with down‐regulated *BjPAT1* expression levels produced considerably more shoot branches than did the control plants (Figure 3b). Normally, bud dormancy breaks 30–35 days after germination in XLH plants under a short‐day photoperiod. In plants with down‐regulated *BjPAT1* expression levels, branches started to appear at 10 days after germination (Figure 3c). Additionally, the T84‐63 plants with down‐regulated *BjPAT1* expression also produced considerably more shoot branches than did the control plants (Figure S6a,b). These results suggested that *BjPAT1* is a negative regulator of shoot branching. A complementation experiment was conducted in which the full‐length *BjPAT1* CDS from the non‐branching line (T84‐63) was expressed in the branching line (XLH) *via* a PTY‐S vector (pTY‐comp‐PAT1). Plants were inoculated with the vector before bud outgrowth initiation. Examinations 10 and 20 days later revealed a lack of obvious bud outgrowth in the plants inoculated with pTY‐comp‐PAT1, unlike in the control plants (Figure 3d,e).

To further confirm the relationship between genotypes and phenotypes, a phenotypic analysis of the BC_3_F_2_ lines of the VC‐029 (non‐branching line) x DHC (branching line) hybridization indicated that BjPAT1 negatively affects shoot branching (Figure 3f–h). Genotyping of the branching, non‐branching, and BC_3_F_2_ lines revealed non‐synonymous SNPs at positions 187 and 388 (Figure 3f). The phenotypes of DHC, VC‐029, and BC_3_F_2_ lines clearly indicated that *BjPAT1* is involved in shoot branching, with BC_3_F_2_ plants producing more branches than either parent (Figure 3g,h).

### PAT1 negatively regulates branching under low R:FR light conditions, suggesting that it influences the shading response

**Figure 4:**
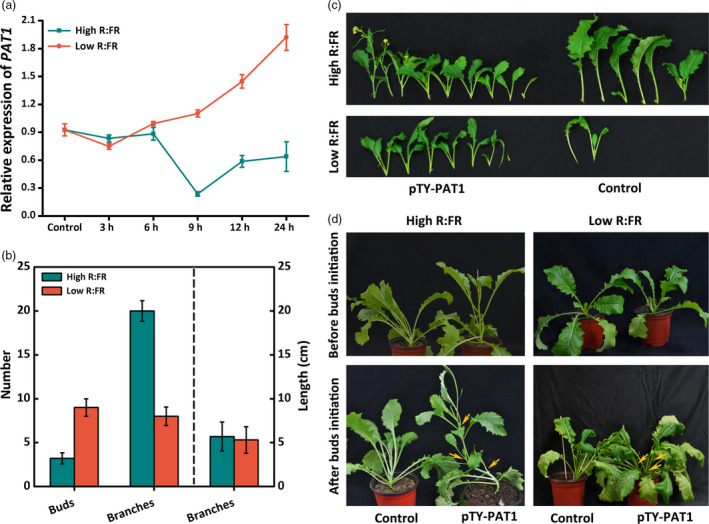
Light regulates *Brassica juncea* shoot branching *via* PAT1. (a) *PAT1* expression patterns in response to different red:far‐red (R:FR) light conditions in XLH plants. (b) Number of branches on XLH plants under high and low R:FR light conditions. (c) Branching phenotypes in pTY‐PAT1 (XLH) plants with down‐regulated *PAT1* expression and control plants under high and low R:FR light conditions. (d) Branching phenotypes in pTY‐PAT1 (XLH) plants with down‐regulated *PAT1* expression and control plants before and after bud initiation under high and low R:FR light conditions.

Under low R:FR light conditions, plants exhibit shade avoidance where PHYA and PHYB play antagonistic roles and bud outgrowth is compromised (Shimizu‐Sato and Mori, 2001). In this study, plants were exposed to red and far‐red light in addition to the background white light to mimic high and low R:FR light conditions. A continuously increased *BjPAT1* expression level was observed under low R:FR light conditions for up to 24 h, while under high R:FR light conditions, the *BjPAT1* expression level was similar to that observed under low R:FR light conditions for 6 h, but then decreased (Figure 4a). There were considerably more branches produced under high R:FR light conditions than under low R:FR light conditions (Figure 4b,c). Additionally, under low R:FR light conditions, plants with down‐regulated *PAT1* expression produced significantly more branches than did control plants (Figure 4c). Low R:FR light conditions are strongly inhibitory towards shoot branching. Our results indicated that under low R:FR light conditions, plants treated with pTY‐PAT1 exhibited greater shoot growth than the control plants, suggesting that *BjPAT1* helps to regulate the low R:FR‐induced inhibition of shoot branching. The same trend was observed for control plants compared with plants with down‐regulated *PAT1* expression levels under relatively high R:FR light conditions (Figure 4c).

Shoot branching generally involves the formation of axillary meristems in the leaf axils and the growth of axillary buds (Shimizu‐Sato and Mori, 2001). We silenced *PAT1* expression before and after bud formation in the branching (XLH) plants to assess whether PAT1 affects the formation of axillary meristems or the initiation of axillary bud outgrowth under two light conditions. Under high R:FR light conditions, the plants that underwent VIGS before buds formed started forming axillary buds at 1 week after treatment, while the plants under low R:FR light conditions lacked buds even after the treatment period was increased to more than 10 days (Figure 4d). Considering the positive effect of red light on bud outgrowth and axillary branching, the treatment was repeated under low R:FR light conditions. We observed that the plants inoculated with pTY‐PAT1 responded in the same way as the control plants. When plants were treated after bud initiation, we detected branching at 1 week after inoculation under high R:FR light conditions, but at 10 days under low R:FR light conditions (Figure 4d). Although low R:FR light conditions inhibit shoot branching, our data indicated that down‐regulated *BjPAT1* expression increased branching under low R:FR light conditions (Figure 4d; Figure S7). The interaction between PAT1 and BRC1 (i.e., putative signal integrator that controls bud outgrowth) suggested that PAT1 affects the shading (low R:FR)‐induced bud outgrowth pathway (Figure S8). Down‐regulated *BRC1* expression was associated with down‐regulated *PAT1* expression under high or low R:FR light treatments (Figure S8). These results indicated that PAT1 helps to break bud dormancy in a BRC1‐dependent manner.

### PAT1 interacts with COL13 to mediate shoot branching and flowering

Plants treated with pTY‐PAT1 flowered earlier than did the control plants (Figure 4c). The early flowering phenotype suggested that there is crosstalk between the PAT1‐regulated branching pathway and the flowering pathway. The screening of a protein–protein interaction database (http://string-db.org) suggested that CONSTANS‐LIKE 13 (COL13) likely interacts with PAT1. A previous study revealed that in *A. thaliana*, COL13 negatively regulates flowering under long‐day conditions, while in rice, it inhibits flowering irrespective of day length (Sheng *et al*., 2016). In this study, we identified a *COL13* (BjuO004978) ortholog in *B. juncea* based on a phylogenetic tree comprising *A. thaliana* and rice *CONSTANS‐LIKE* genes (Figure S9). Moreover, down‐regulated *COL13* expression induced early flowering in *B. juncea*, indicating that it is a conserved negative regulator of flowering (Figure S10).

**Figure 5:**
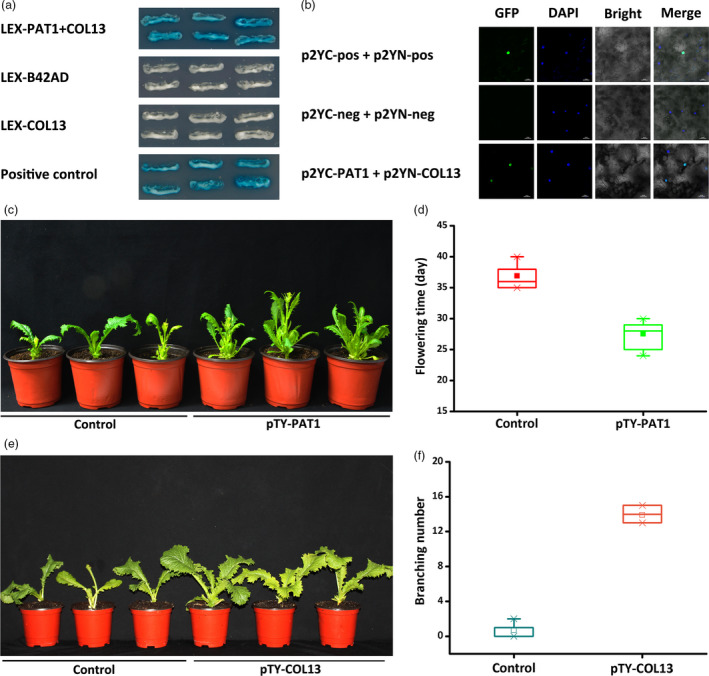
Interaction between PAT1 and COL13 regulates shoot branching and flowering in *Brassica juncea*. (a) Interaction between PAT1 and COL13 determined by yeast two‐hybrid assay. (b) Interaction between PAT1 and COL13 determined by bimolecular fluorescence complementation. (c and d) Analysis of flowering time in pTY‐PAT1 plants with down‐regulated *PAT1* expression and control (XLH) plants. (e and f) Number of branches on pTY‐COL13 plants with down‐regulated *COL13* expression and control (XLH) plants.

**Figure 6:**
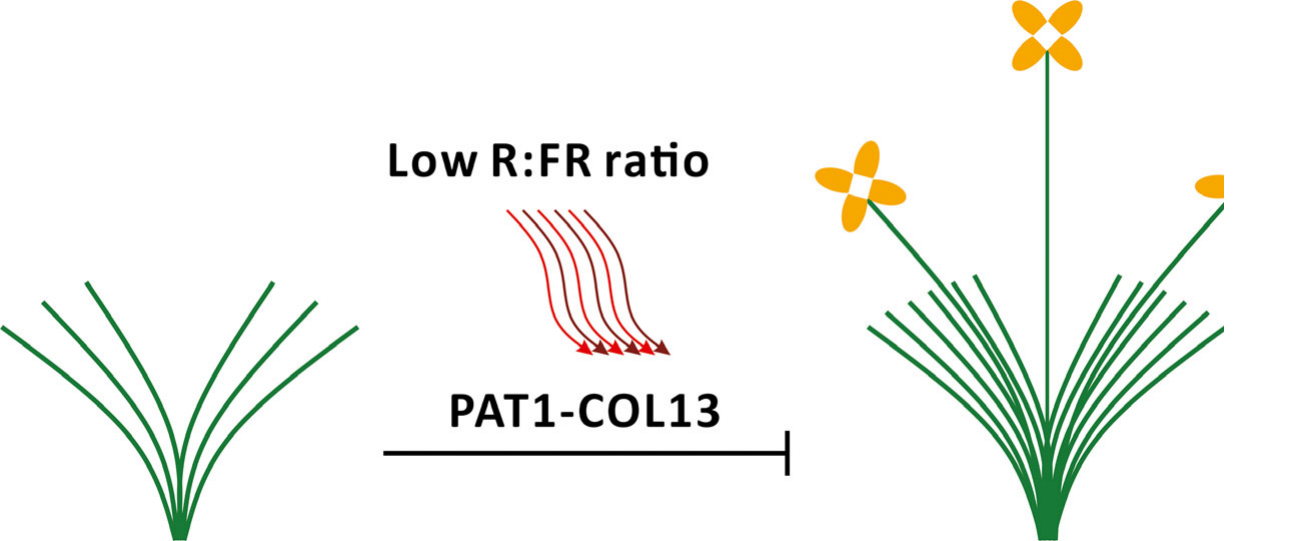
Proposed working model in which PAT1 interacts with COL13 to orchestrate shoot branching and flowering. Green lines represent shoot branches. Red and purple wavy lines represent red (R) and far‐red (FR) light, respectively.

We conducted a yeast two‐hybrid assay and a bimolecular fluorescence complementation analysis to examine the interaction between PAT1 and COL13. The co‐transformed yeast cells expressing *PAT1* and *COL13* activated the expression of the *LacZ* reporter gene, suggesting that PAT1 interacts with COL13 *in vitro* (Figure 5a). The bimolecular fluorescence complementation analysis confirmed the interaction between PAT1 and COL13 *in vivo* (Figure 5b). To further verify the relationship between PAT1 and COL13, we analysed *BjCOL13* expression in plants treated with pTY‐PAT1 as well as *BjPAT1* expression in plants treated with pTY‐COL13. The results demonstrated that *COL13* expression is directly regulated by *PAT1* expression during flowering in *B. juncea*. Additionally, we detected down‐regulated *BjCOL13* expression in plants with down‐regulated *BjPAT1* expression. Meanwhile, plants with down‐regulated *BjCOL13* expression levels showed decreased *PAT1* expression levels (Figure S11). Consequently, plants with down‐regulated *PAT1* expression flowered earlier than did the control plants (Figure 5c,d). Plants with down‐regulated *BjCOL13* expression produced more branches than did control plants (Figure 5e,f). We also assessed the effects of COL13 on branching in another non‐branching line (EU07). Our analyses suggested that branching was induced in plants with down‐regulated *BjCOL13* expression (Figure S12). Thus, we developed a working model in which PAT1 interacts with COL13 to mediate shoot branching and flowering (Figure 6).

## Discussion

Plant shoot architecture largely determines leaf, flower, and fruit production as well as light capture efficiency, reproductive success, and yield, and is controlled by numerous endogenous and environmental factors, including hormones and light (Kebrom *et al*., 2013). The results of our study revealed the effects of PAT1 on shoot branching and flowering in leafy *B. juncea*. The *PAT1* gene, which encodes a signal transduction factor in the PHYA pathway, belongs to the GRAS family and is strongly related to plant responses to low R:FR light conditions (Bolle *et al*., 2000; Torres‐Galea *et al*., 2013). The genes in the *PAT1* branch of the GRAS family regulate diverse functions in plants. Several *A. thaliana* genes belong to the *AtPAT1* sub‐branch of the GRAS family, namely *AtSCL21, AtSCL5, AtSCL13*, and *AtSCL1* (Yuan *et al*., 2016). Two rice genes, *OsCIGR1* and *OsCIGR2*, are grouped in the same sub‐branch. The expression of these genes is rapidly induced in suspension‐cultured *Oryza sativa* cells after the N‐acetylchitooligosaccharide elicitor is perceived and during co‐cultivation with the rice blast fungus (Day *et al*., 2003). Additionally, an investigation of tomato plants indicated that *SlGRAS2* and *SlGRAS3* transcript levels are highest in the stem and in response to auxin, implying that these genes are likely involved in vegetative development (Zhou *et al*., 2011). The GRAS family of transcription factors also includes genes encoding MONOCULM1 (MOC1) and Lateral Suppressor (LS, LAS) in rice, tomato, and *A. thaliana* (Greb *et al*., 2003; Li *et al*., 2003; Schumacher *et al*., 1999). Moreover, MOC1, the first tillering regulator characterized in rice, controls the initiation and outgrowth of axillary meristems during the vegetative and reproductive stages. A loss‐of‐function mutation to rice *MOC1* reportedly decreases the number of tillers and panicle branches (Li *et al*., 2003). Unlike other members of the same family, the diverse functions of *PAT1* related to shoot branching had not been clarified. In this study, we identified a *B. junc*ea *PAT1* gene among five homologs as a potential regulator of shoot branching. Experiments involving gene expression analyses, silencing of *PAT1* (BjuB019592) expression, and functional analyses under different light conditions confirmed that *PAT1* mediates branching. The genotyping of branching and non‐branching lines detected three non‐synonymous SNPs corresponding to positions 8, 63, and 130 of the encoded amino acid sequence. These SNPs may be responsible for the disruption of protein function during branching. Silencing of *PAT1* expression induced the production of branches in branching and non‐branching lines of leafy *B. juncea*, confirming that PAT1 helps to regulate branching.

Light intensity and quality affect shoot branching through plant shade avoidance responses, which reflect the competition among plants for light. Low light intensity and increased exposure to far‐red light inhibit bud outgrowth, ultimately resulting in fewer branches (Wang *et al*., 2018). Low R:FR light conditions stimulate developmental changes that enable plants to withstand the impending competition for resources. Phytochromes absorb light in the red and far‐red regions of the spectrum, and *A. thaliana* are encoded by a five‐member gene family (*PHYA* to *PHYE*), with each member existing in two photo‐interconvertible forms (Pr and Pfr). Moreover, PHYB is crucial for detecting low or high R:FR light conditions (Ballare *et al*., 1990, 1997), and a loss‐of‐function mutation to PHYB in *A. thaliana* and *Sorghum bicolor* results in arrested bud outgrowth (Finlayson *et al*., 2010; Kebrom *et al*., 2006). The antagonistic relationship between PHYA and PHYB has been debated for a long time. A recent study concluded that PHYA negatively regulates auxin responses, but that study focused on the role of auxin during hypocotyl elongation (Yang *et al*., 2018a). Other studies on pea and cotton confirmed that PHYA inhibits shoot branching (Abdurakhmonov *et al*., 2014; Weller *et al*., 1997). As a positive regulator of PHYA, PAT1 was expected to be a major contributor to the inhibition of shoot branching. Our hypothesis was confirmed by the *PAT1* expression pattern under low and high R:FR light conditions, the results of a VIGS analysis under different light conditions, and differences in the number of branches between control plants and *PAT1‐*knockdown plants. Our data suggest that PAT1 influences shading (low R:FR)‐induced bud outgrowth.

Branch development can be separated into two stages; bud formation and bud outgrowth (Kebrom *et al*., 2013; Wang *et al*., 2018). Axillary buds arise in leaf axils and either grow or enter dormancy in a process that is coordinated at the whole‐plant level. This process is related to apical dominance, which involves genes related to the transition from vegetative to reproductive growth (Wang *et al*., 2018). Our data suggest that PAT1 helps to break bud dormancy, rather than contribute to meristem formation, under low R:FR light conditions. Bud outgrowth displays a plasticity that helps the plant match the degree of shoot development to resource availability, thus maximizing adaptation to changing environmental and endogenous conditions (Martin‐Fontecha *et al*., 2018). This response may have played a critical evolutionary role during plant colonization of habitats with seasonal climate fluctuations.

The indeterminate nature of *B. juncea* helps the plant undergo vegetative growth and shoot branching simultaneously. However, inflorescence branching occurs at a later plant development stage along with the continuation of shoot branching (Kaur and Banga, 2015). The transition from vegetative to reproductive growth is strictly controlled by two parallel pathways involving CO/FT as positive regulators of early flowering and TFL1, which is similar to FT, as a strong inhibitor of early flowering (Bohlenius *et al*., 2006). The observed early flowering in the plants treated with pTY‐PAT1 compelled us to conduct additional experiments analysing the relationships between *PAT1* and genes controlling flowering. Our protein–protein interaction analysis confirmed the physical interaction between PAT1 and COL13. Being a long‐day plant, *B. juncea* favors a short photoperiod for vegetative growth. However, the results of *COL13‐*knockdown experiments revealed early flowering even under short‐day conditions, consistent with a previous report regarding TFL1 (Hanano and Goto, 2011). In non‐branching knockdown plants, increased branching was observed under short‐day conditions, while control plants remained in the non‐branching state. Moreover, in the branching line, early branching occurred, but the plants produced fewer branches than did plants treated with pTY‐PAT1. The results of our *COL13* VIGS analysis are similar to those of a previous analysis of TFL1 under short‐day conditions. In the earlier study on *A. thaliana* TFL1, a similar rosette leaf phenotype was observed in the mutant plants (Hanano and Goto, 2011). These results were consistent with those of a previous study on rice under short‐day conditions (Sheng *et al*., 2016), except for the observed increase in shoot branching. Among *A. thaliana CONSTANS‐LIKE* genes, *COL7* reportedly regulates branching and the shade avoidance response involving the PHYB‐mediated pathway, as well as flowering (Wang *et al*., 2013; Zhang *et al*., 2014). Additionally, *COL12* overexpression in *A. thaliana* affects plant architecture by increasing the number of rosette branches and decreasing inflorescence height (Ordonez‐Herrera *et al*., 2018). CONSTANS‐LIKE 3 is a positive regulator of red‐light signalling and root growth in *A. thaliana* (Datta *et al*., 2006). These findings imply that crosstalk between flowering and phytochrome pathways regulates plant architecture. The results described herein suggest that the interaction between PAT1 and COL13 regulates branching and flowering in a process that depends on the PHYA pathway.

In conclusion, we revealed that PAT1 and COL13 coordinately function as negative regulators of shoot branching and flowering in leafy *B. juncea*. The alleviation of low R:FR‐induced inhibition of branching implies that PAT1 is involved in the shade response of plants. The non‐synonymous substitutions in *PAT1* provide new targets for genome editing research aimed at improving crop productivity and enabling an earlier harvest. Furthermore, the effects of PAT1 on bud outgrowth suggest its potential application for plant regeneration.

## Experimental procedures

### Plant materials and analysis of branching

*Brassica juncea* var. *multiceps* (XLH), a branching line, and *Brassica juncea* var. *tumida* (T84‐63), a non‐branching line, were crossed to generate F_1_ and F_2_ populations for phenotypic and genetic analyses. The number of branches was recorded for 20 individual XLH, T84‐63, and F_1_ plants, and 236 individual F_2_ plants. The F_2_ population from the cross between XLH and T84‐63 was used for a bulked segregant analysis by resequencing. A branching line (DHC) and a non‐branching line (VC029) were selected to construct a backcrossing line (BC3F2) using DHC as the recurrent parent for genotyping and phenotyping analyses. Branching lines (03C1110, 03C1113, V03A0066, V03A0067) and non‐branching lines (84‐66, AU213, and IN30) were used for genotyping analyses of PAT1.

### Bulked segregant analysis by resequencing

Branching and non/less‐branching plants (35 plants each) were selected from the F_2_ population of the cross between XLH and T84‐63 for a bulked segregant analysis. Total DNA was extracted from each plant and mixed to form the branching and non/less‐branching pools. The bulked DNA samples were digested with *HindIII‐HF*, *NdeI*, and *MseI*, and the resulting DNA fragments (380–430 bp) were analysed with a specific‐locus amplified fragment (SLAF) sequencing strategy involving the Illumina HiSeq 2500 system according to an Illumina protocol (Sun *et al*., 2013). Filtered clean reads were aligned to the reference genome (version 1.5) (Yang *et al*., 2016) using the Burrows–Wheeler Aligner program (Li and Durbin, 2009). Single nucleotide polymorphisms were identified using the GATK toolkit (McKenna *et al*., 2010) and SAMtools (Li *et al*., 2009) for each pool and between pools based on the reference genome. The SNPs with integrity ≥50% and minor allele frequency ≥5% were analysed further. The SNPs linked to branching were then identified *via* an association analysis involving Euclidean Distance (Hill *et al*., 2013). Regression and fitting analyses of the Euclidean Distance for SNP markers on the same chromosome were used to determine the threshold Euclidean Distance (0.1184). Candidate genes in associated regions were annotated using the nr, Swiss‐Prot, GO, COG, and KEGG databases.

### Phylogenetic analysis and genotyping of *PAT1*

We obtained 33 *A. thaliana* GRAS transcription factor genes from the Arabidopsis Gene Family Information database of The Arabidopsis Information Resource. After aligning *PAT1* (BjuB019592), its homoeolog (BjuA008320), and other GRAS transcription factor genes with the Clustal Omega program, a neighbour‐joining phylogenetic tree was constructed with MEGA 5.0 (1000 bootstrap replicates). We also PCR‐amplified the full‐length *PAT1* CDSs from several branching lines (03C1110, 03C1113, V03A0066, and V03A0067) and non‐branching lines (T84‐66, AU213, and IN30) to further analyse the SNPs. Details of the PCR primers are listed in Table S8.

### Functional analysis of *PAT1* and *COL13*

A TYMV‐based VIGS system was used to functionally characterize *PAT1* and *COL13* as previously described (Pflieger *et al*., 2008; Shopan *et al*., 2017). The pTY vector was digested with *SnaBI*, and the resulting linearized vector was analysed by gel electrophoresis to confirm specificity. Additionally, we designed an 80‐nt palindromic oligonucleotide sequence specific to BjuB019592 to avoid down‐regulation of any *PAT1* homologs. The self‐hybridized 40‐nt sequence was ligated using a T4 DNA ligase system (Clontech, Palo Alto, CA). The amplification of a TYMV‐CP gene of the expected size (520 nt) was used to identify positive clones. Details of the pTY‐CP primer pairs are provided in Table S8. For the virus infiltration, 5 μg purified pTY‐S carrying the target gene was diluted in 25 μL ddH_2_O, which was then used to infiltrate 2–4 fully expanded leaves from XLH plants. The control plants were infiltrated with the empty pTY‐S vector. The pTY‐PDS‐IR vector, which was used as a positive control, causes photo‐bleaching because of the associated silencing of the phytoene desaturase gene. Infiltrated plants were incubated in a growth chamber set at 22 °C (day)/20 °C (night) with an 8‐h light/16‐h dark cycle. Genomic DNA was extracted from plant leaves using the DNeasy kit (Qiagen, Hilden, Germany) for a subsequent PCR amplification with the pTY‐CP forward and reverse primers to confirm the presence of pTY‐PAT1 in plants.

Two non‐branching lines (T84‐63 and EU07) and one branching line (XLH) were used to functionally analyse *COL13* (BjuO004978). A pTY‐VIGS system was used to knock‐down the gene. Additionally, an 80‐nt palindromic oligonucleotide sequence was used to construct the pTY‐COL13 vector. Plants were inoculated with the virus at 10 days after germination. The delayed‐flowering control plants and treated plants were then incubated under short‐day conditions (8‐h light/16‐h dark) for 3 weeks, after which gene expression levels were analysed by quantitative real‐time (qRT)‐PCR assay (Applied Biosystems, Foster City, CA). The *COL13* and *PAT1* expression levels in the aforementioned lines were analysed. The number of branches and flowering time were recorded and compared between the control and treated plants. At least 10 plants were collected as biological replications under the same short‐day photoperiod to control early branching and flowering.

To express *BjPAT1* from a non‐branching line (T84‐63) in a branching line (XLH) with the TYMV‐derived pTY‐S vector, the full‐length BjuB019592 CDS from T84‐63 was amplified by PCR using a forward primer with a *SmaI* restriction enzyme site and a reverse primer with an *AgeI* restriction enzyme site. The amplified product was cloned into the pTY‐S virus‐derived vector containing the CaMV 35S promoter with the T4 DNA ligase system (Clontech) to generate pTY‐S‐PAT1. The pTYCP‐F (TCCACCCTCACCACCTTC) and pTYCP‐R (CCCTAATTCCCTTATCTGGG) primer pair was used to confirm the presence of *BjPAT1* in the clone. The TYMV isolation and inoculation procedures were completed as described above. Plants were inoculated before buds formed. The inoculated plants and control plants were exposed to the same light conditions and photoperiod.

### Red and far‐red light treatment

*Brassica juncea* plants from the branching line (XLH) were grown under white light under short‐day conditions (8‐h light/16‐h dark). To check the transient response of *PAT1*, plants at the bud arrest stage were exposed to low (0.1–0.3) and high (1.6) R:FR light conditions for 24 h in growth chambers. Simulated shade (white and far‐red lights) conditions were generated by enriching the white light with supplementary far‐red light provided by LED lamps (www.quantumdev.com or www.philips.com/horti). The R:FR ratio was calculated based on 30‐nm ranges around the red (640–670 nm) and far‐red (720–750 nm) peaks. For light‐induced VIGS, XLH plants were first grown under white light. At the 2–3‐leaf stage, plants were treated with red, far‐red, and normal white light conditions to stimulate responses to low and high R:FR light conditions. To induce the pTY‐PAT1 response under red and far‐red lights, plants were first grown under white light and short‐day conditions. At 24 h after inoculation with pTY‐PAT1, all plants were treated with red and far‐red lights under short‐day conditions in growth chambers.

### Yeast‐two‐hybrid assay

A yeast‐two‐hybrid assay was conducted with the LexA system as previously described. To generate the LexA‐PAT1 and LexA‐COL13 vector constructs, the CDSs of the genes were amplified by PCR with the primer pairs listed in Table S8. The PCR products were then cloned into the *MfeI‐XhoI* sites of the pLexA vector (Clontech). To generate the *AD‐COL13* construct (for the LexA yeast two‐hybrid system), the *COL13* CDS was amplified by PCR with the primer pairs listed in Table S8, and then cloned into the *MfeI‐XhoI* sites of the pB42AD vector (Clontech).

For LexA‐based yeast two‐hybrid assays, cells of the yeast strain EGY48 harboring the reporter plasmid *p8op:LacZ* (Clontech) were co‐transformed with the LexA and AD fusion plasmids. For plate assays, transformants were grown on agar‐solidified SD/−Ura/−His/−Trp dropout medium containing X‐gal (5‐bromo‐4‐chloro‐3‐indolyl‐β‐d‐galactopyranoside). Positive transformants were stained blue on this medium. Yeast cells were transformed as described in the Yeast Protocols Handbook (Clontech).

### Bimolecular fluorescence complementation analysis

Bimolecular fluorescence complementation vectors p2YN (amino acids 1–158) and p2YC (amino acids 159–238) were used to generate the N‐ and C‐termini of PAT1 and COL13 (OARDC, Ohio State University, Wooster, OH, USA). Specifically, the full‐length *PAT1* and *COL13* CDSs lacking a stop codon were PCR‐amplified to introduce *PacI* and *SpeI* restriction enzyme sites. The bimolecular fluorescence complementation vectors and PCR‐amplified fragments were digested with *PacI* and *SpeI* (New England Biolabs, Beverly, MA) and then ligated with the T4 DNA ligase system (Clontech). The resulting vectors were used to transform *Agrobacterium tumefaciens* strain GV3101 cells, which were then grown in liquid cultures that were used to infiltrate *Nicotiana benthamiana* leaves. The fluorescence from the yellow fluorescent protein was detected and photographed under a confocal laser scanning microscope at 3 days after infiltration (Siriwardana and Lamb, 2012).

### Quantitative real‐time PCR

Total RNA was extracted from samples using the RNeasy Plant Mini Kit (Qiagen) and then digested with RNase‐free DNase (Qiagen). The total RNA (1 μg) was used as the template to synthesize cDNA with a Reverse Transcriptase M‐MLV kit (Takara, Otsu, Japan). The expression levels of selected genes were analysed by qRT‐PCR, which was completed with the StepOne system (Applied Biosystems). All analyses were conducted with three biological replicates. Details of the primers used in this study are listed in Table S8.

## Supporting information


**Figure S1** Distribution of SLAF‐tags and SNPs on chromosomes.
**Figure S2** Annotation of *Brassica juncea* genes linked to branching based on association analysis.
**Figure S3** Alignment of *PAT1* (BjuB019592) cDNA sequences from branching (XLH) and non‐branching (T84‐63) *Brassica juncea* lines.
**Figure S4** *PAT1* expression in control *Brassica juncea* XLH plants and after gene silencing with pTY‐PAT1.
**Figure S5** Expression levels of *PAT1* homoeologous and homologous genes in *Brassica juncea* XLH plants after gene silencing with pTY‐PAT1.
**Figure S6** Branching phenotypes in control plants and pTY‐PAT1 plants (non‐branching line: T84‐63) with down‐regulated *PAT1* expression.
**Figure S7** Number of branches in pTY‐PAT1 line (XLH) with down‐regulated *PAT1* expression and control under high and low R:FR conditions.
**Figure S8** Interaction between BRC1 and PAT1 and analysis of *BRC1* expression.
**Figure S9** Phylogenetic tree of *CONSTANS‐like* genes, including *BjCOL13* (BjuO004978) and *COL* genes from *Arabidopsis thaliana* and rice. Round black dot indicates *BjCOL13* gene.
**Figure S10** Flowering phenotypes of pTY‐COL13 plants (non‐branching lines: a, T84‐63; b, EU07) with down‐regulated *COL13* expression and control plants.
**Figure S11** Comparison of *COL13* and *PAT1* expression patterns in control, pTY‐PAT1, and pTY‐COL13 plants.
**Figure S12** Branching phenotype of pTY‐COL13 plants (non‐branching line: EU07) with down‐regulated *COL13* expression and control plants.
**Table S1** Analysed data and normal distribution test results for branching in *Brassica juncea* F_2_ population
**Table S2** SLAF‐sequencing of branching and non‐branching *Brassica juncea* F_2_ bulks
**Table S3** SLAF‐tag and SNP data for branching and non‐branching *Brassica juncea* F_2_ bulks
**Table S4** Data for SLAF‐tags and SNPs on chromosomes
**Table S5** Genomic regions associated with branching in *Brassica juncea*
**Table S7** Annotation of branching candidate genes linked to SNPs
**Table S8** Details of primers used in this study

**Table S6** Annotation of candidate genes associated with branching in *Brassica juncea*
