# Supplementary material for: Phytochrome A signal transduction 1 and CONSTANS‐LIKE 13 coordinately orchestrate shoot branching and flowering in leafy Brassica juncea
Source: Plant Biotechnol J. 2019 Feb 20;17(7):1333–43. doi: 10.1111/pbi.13057 (PMC6576096; doi:10.1111/pbi.13057)
Supplement: Supplementary file 1 — Figure S1 Distribution of SLAF‐tags and SNPs on chromosomes. Figure S2 Annotation of Brassica juncea genes linked to branching based on association analysis. Figure S3 Alignment of PAT1 (BjuB019592) cDNA sequences from branching (XLH) and non‐branching (T84‐63) Brassica juncea lines. Figure S4 PAT1 expression in control Brassica juncea XLH plants and after gene silencing with pTY‐PAT1. Figure S5 Expression levels of PAT1 homoeologous and homologous genes in Brassica juncea XLH plants after gene silencing with pTY‐PAT1. Figure S6 Branching phenotypes in control plants and pTY‐PAT1 plants (non‐branching line: T84‐63) with down‐regulated PAT1 expression. Figure S7 Number of branches in pTY‐PAT1 line (XLH) with down‐regulated PAT1 expression and control under high and low R:FR conditions. Figure S8 Interaction between BRC1 and PAT1 and analysis of BRC1 expression. Figure S9 Phylogenetic tree of CONSTANS‐like genes, including BjCOL13 (BjuO004978) and COL genes from Arabidopsis thaliana and rice. Round black dot indicates BjCOL13 gene. Figure S10 Flowering phenotypes of pTY‐COL13 plants (non‐branching lines: a, T84‐63; b, EU07) with down‐regulated COL13 expression and control plants. Figure S11 Comparison of COL13 and PAT1 expression patterns in control, pTY‐PAT1, and pTY‐COL13 plants. Figure S12 Branching phenotype of pTY‐COL13 plants (non‐branching line: EU07) with down‐regulated COL13 expression and control plants. Table S1 Analysed data and normal distribution test results for branching in Brassica juncea F2 population Table S2 SLAF‐sequencing of branching and non‐branching Brassica juncea F2 bulks Table S3 SLAF‐tag and SNP data for branching and non‐branching Brassica juncea F2 bulks Table S4 Data for SLAF‐tags and SNPs on chromosomes Table S5 Genomic regions associated with branching in Brassica juncea Table S7 Annotation of branching candidate genes linked to SNPs Table S8 Details of primers used in this study [file PBI-17-1333-s002.docx]

Table S1 Analyzed data and normal distribution test results for the branching in the *B. juncea* F_2_ population

| **Trait** | **Mean** | **Variance** | **SD** | **CV** | **t-distribution quantiles** | **Critical value of Z** |
| --- | --- | --- | --- | --- | --- | --- |
| Branching | 20.37 | 37.07 | 6.09 | 0.30 | 1.97 | 3.65 |

Average of branching number of XLH: 23.3

Average of branching number of T84-63: 0

Average of branching number of F_1_: 25.9

SD: Standard Deviation

CV: Coefficient of Variation

Table S2 SLAF-sequencing of branching and non-branching *Brassica juncea* F_2_ bulks

| **DNA pool (number of plants)** | **Total reads** | **Q30 (%)** | **GC (%)** | **Total map (%)** | **Properly mapped (%)** |
| --- | --- | --- | --- | --- | --- |
| Branching bulk (35) | 6,496,349 | 70.02 | 43.19 | 76.49 | 62.90 |
| Non-branching bulk (35) | 6,997,371 | 63.26 | 42.69 | 71.60 | 57.72 |

Total map: the ratio of reads mapped to genome to all sequencing reads

Properly mapped: the ration of reads pair-end to mapped to genome all sequencing reads

Table S3 SLAF-tag and SNPs data for branching and non-branching *Brassica juncea* F_2_ bulks

| **DNA pool (number of plants)** | **SLAF number** | **Total depth** | **Average depth** | **Total SNP** | **SNP number** | **Heter ratio (%)** |
| --- | --- | --- | --- | --- | --- | --- |
| Branching bulk (35) | 73,067 | 3,858,208 | 52.80 | 40,842 | 32,760 | 22.48 |
| Non-branching bulk (35) | 73,165 | 3,821,481 | 52.23 | 40,842 | 33,228 | 23.85 |

Total depth: total reads number of SLAF-tags

Average depth: average reads number on each SLAF-tag

SNP number: SNP number in each bulks

Heter ratio: the ratio of heterozygous SNP

Table S4 Data regarding of SLAF-tags and SNPs on chromosomes

| **Chromosome ID** | **SLAF number** | **SNP number** |
| --- | --- | --- |
| J01 | 3,250 | 2,481 |
| J02 | 3,037 | 1,914 |
| J03 | 3,618 | 1,661 |
| J04 | 2,004 | 7,99 |
| J05 | 2,647 | 1,823 |
| J06 | 2,635 | 2,266 |
| J07 | 2,583 | 1,459 |
| J08 | 2,168 | 1,393 |
| J09 | 4,381 | 2,900 |
| J10 | 1,772 | 1,066 |
| J11 | 3,732 | 1,891 |
| J12 | 5,059 | 2,081 |
| J13 | 4,533 | 1,875 |
| J14 | 3,544 | 1,476 |
| J15 | 4,657 | 2,437 |
| J16 | 3,066 | 1,411 |
| J17 | 3,327 | 1,805 |
| J18 | 4,971 | 2,722 |
| Others | 12,401 | 7,382 |
| Total | 73,393 | 40,842 |

Table S5 Genomic regions associated with branching in *Brassica juncea*.

| **Chromosome** | **Start** | **End** | **Size (Mb)** | **Gene number** |  |
| --- | --- | --- | --- | --- | --- |
| J03 | 14,292 | 10,118,114 | 10.1 | 1,495 |  |
| J11 | 108,765 | 7,576,494 | 7.47 | 859 |  |
| Total | - | - | - | 2,354 |  |

Table S7 Annotation of branching candidate genes linked to SNP (43,944,905-44,086,951)

| **Gene ID** | **location** | **Annotation** |
| --- | --- | --- |
| *BjuB019588* | 43951163-43952125 | Myb-like DNA-binding domain transcription factor LUX |
| *BjuB019589* | 43953714-43955125 | UDP-glycosyltransferase 76E12 |
| *BjuB019590* | 43957084-43957575 | UDP-glycosyltransferase 76E5 |
| *BjuB019591* | 43958593-43961574 | Retroviral aspartyl protease |
| *BjuB019592* | 43967166-43968009 | Scarecrow-like transcription factor PAT1 |
| *BjuB019593* | 43983192-43984016 | Predicted glutathione S-transferase T3-like |
| *BjuB019594* | 43999843-44000007 | pseudogene |

Table S8 Details regarding the primers used in this study

| **Gene** | **Forward primer (5′-3′)** | **Reverse primer (5′-3′)** |
| --- | --- | --- |
| *PAT1-CDS* | ATGTTGGCGGGAGGAGGAG | TCATTTCCATGCACCGGAGATGAC |
| *PAT1-qPCR* | TGACGGAGGAGAAGAGGTGT | TAACCGGTCACTCCGCTC |
| *pTY-PAT1* | ATGAGCTTCTAGGGAAATGGAGGTCACGGTTTGAGATGGCGCCATCCCAAACCGTGACCTCCATTTCTCTAGAAGCTCA | |
| *COL13-CDS* | ATGGAAAAAGAAACCGAAGA | TTATGGATGTGACTTAGCGAA |
| *COL13-qPCR* | ACAAGGTTGACGACGACACA | ACTTAGCGAAACGTCCCCTG |
| *pTY- COL13* | TTGACGACGACACATGAGATCAACAGTCTTGAGAGAAACTAGTTTCTCTCAAGACTGTTGATCTCATGTGTCGTCGTCAA | |
| *BjuA008320-qPCR* | ACATGAGCTTGAGCTTTTCTTGGA | TCCCAACAGAGTTGGCAGGC |
| *BjuB042895-qPCR* | GCCAGCCAACTCTGATGGGA | TCCGTCACACAAGAGCCCGA |
| *BjuB044594-qPCR* | CGGTATTGCGTGCTTGAGCC | TGGCCCCATCATCACTGTCT |
| *BjuB007739-qPCR* | AGGCCAACTCTGTTGGGAAGC | TCGTCCGTCACACAAGAGGC |
| *BRC1-qPCR* | AGCAGAACGGATCGGCACAG | CGGGTTTGGCTTGTGTGAGC |
| *PAT1-Y2H* | GAATTCATGTTGGCGGGAGGAGGAG | GTCGACTCATTTCCATGCACCGGAGATGAC |
| *COL13-Y2H* | GAATTCATGGAAAAAGAAACCGAAGA | GTCGACTTATGGATGTGACTTAGCGAA |
| *Actin-qPCR* | CGGTTCCTCTCGTACTAGGTTGA | CCGTCGTGAGACAGGTTAGTTTT |


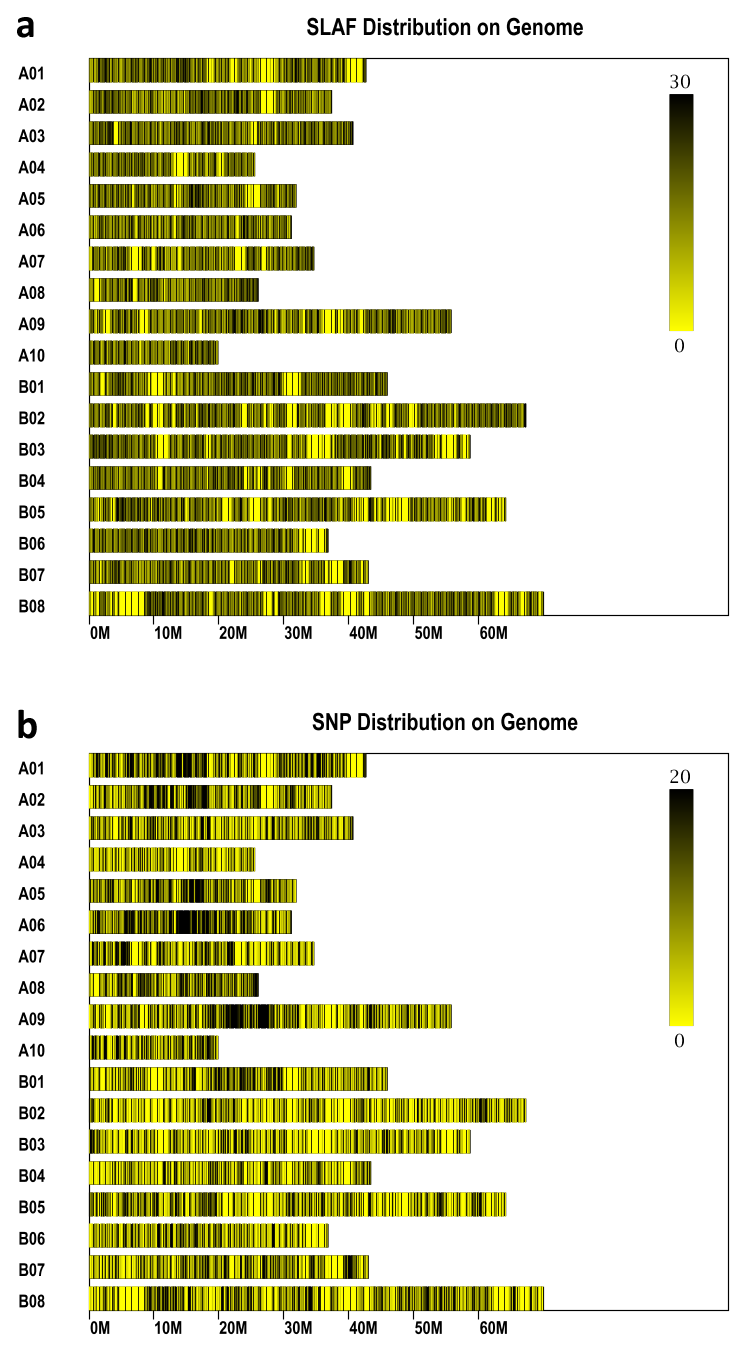


**Figure S1** Distribution of SLAF-tags and SNPs on chromosomes. a, SLAF-tags on chromosomes. b, SNPs on chromosomes.


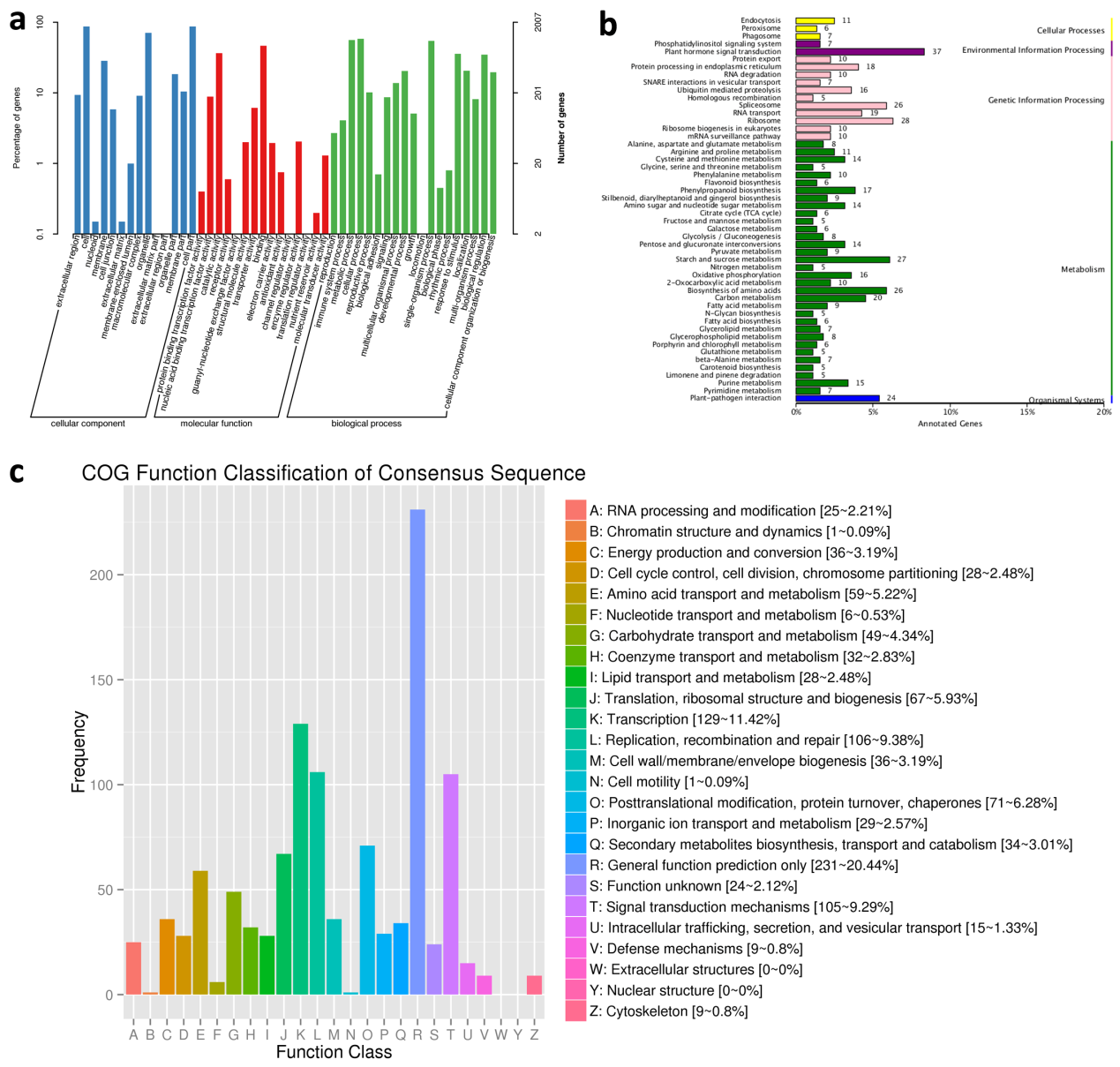


**Figure S2** Annotation of *B. juncea* genes linked to branching based on an the association analysis. a, Gene Ontology (GO) analysis. b, Kyoto Encyclopedia of Genes and Genomes (KEGG) pathway enrichment analysis. b, Cluster of Orthologous Groups (COG) analysis.


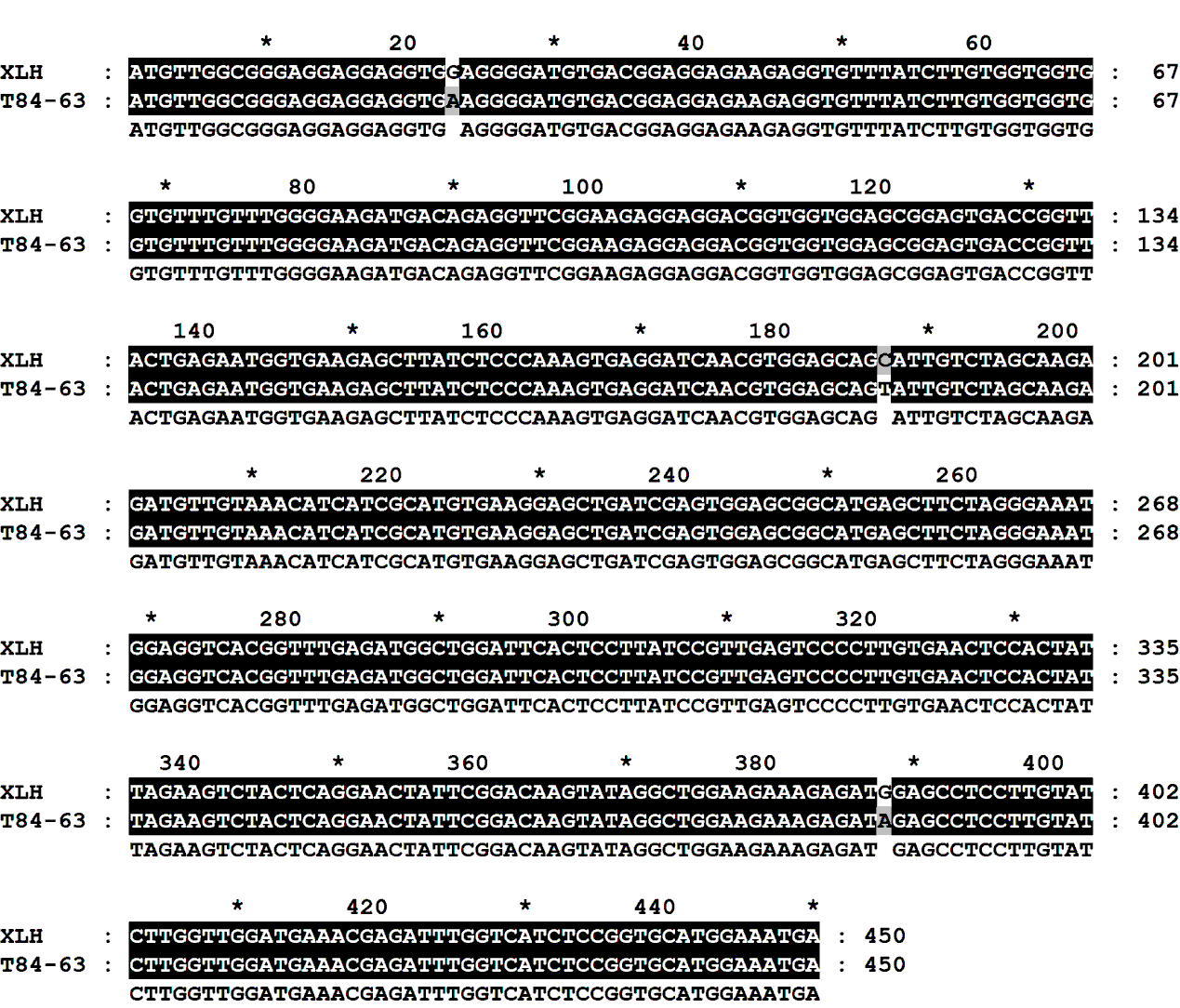


**Figure S3** Alignment of *PAT1* (*BjuB019592*) cDNA sequences from branching (XLH) and non-branching (T84-63) *B. juncea* lines.





**Figure S4** *PAT1* expression in control *B. juncea* XLH plants and following gene silencing with pTY-PAT1.

**

**

**Figure S5** Expression levels of *PAT1* homoeologous and homologous genes in *B. juncea* XLH plants following gene silencing with pTY-PAT1.


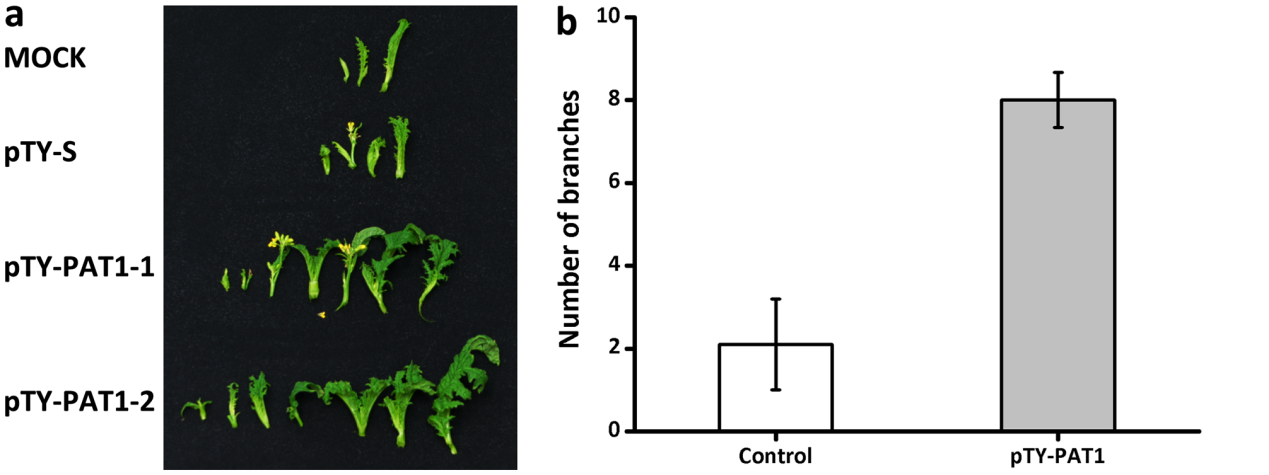


**Figure S6** Branching phenotypes in control plants and the pTY-PAT1 plants (non-branching line: T84-63) with down-regulated *PAT1* expression. **a,** Isolated branches. **b,** Statistic number of branches.





**Figure S7** Branches numbers of in pTY-PAT1 line (XLH) with down-regulated expression of *PAT1* and control under high and low R:FR condition.


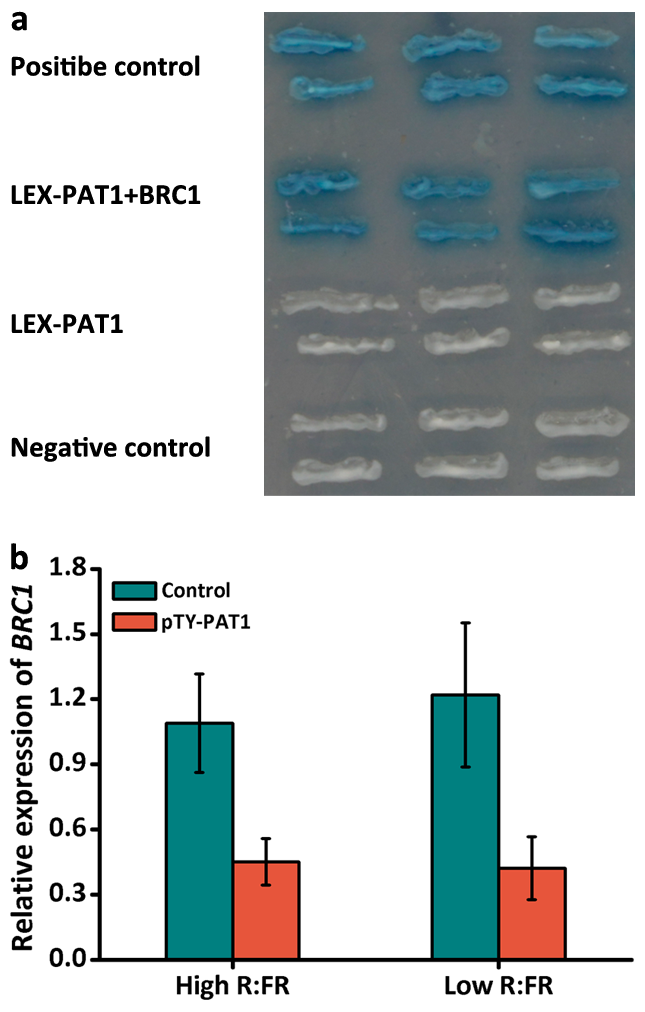


**Figure S8** Interaction between BRC1 and PAT1 and analysis of *BRC1* expression. **a,** Interaction between BRC1 and PAT1 in a yeast-two-hybrid assay. **b,** *BRC1* expression in control *B. juncea* XLH plants and following gene silencing with pTY-PAT1 under high and low red:far-red light conditions.





**Figure S9** Phylogenetic tree of *CONSTANS-like* genes, including *BjCOL13* (BjuO004978) and *COL* genes from *Arabidopsis thaliana* and rice. The *BjCOL13* gene is indicated with a round black dot.


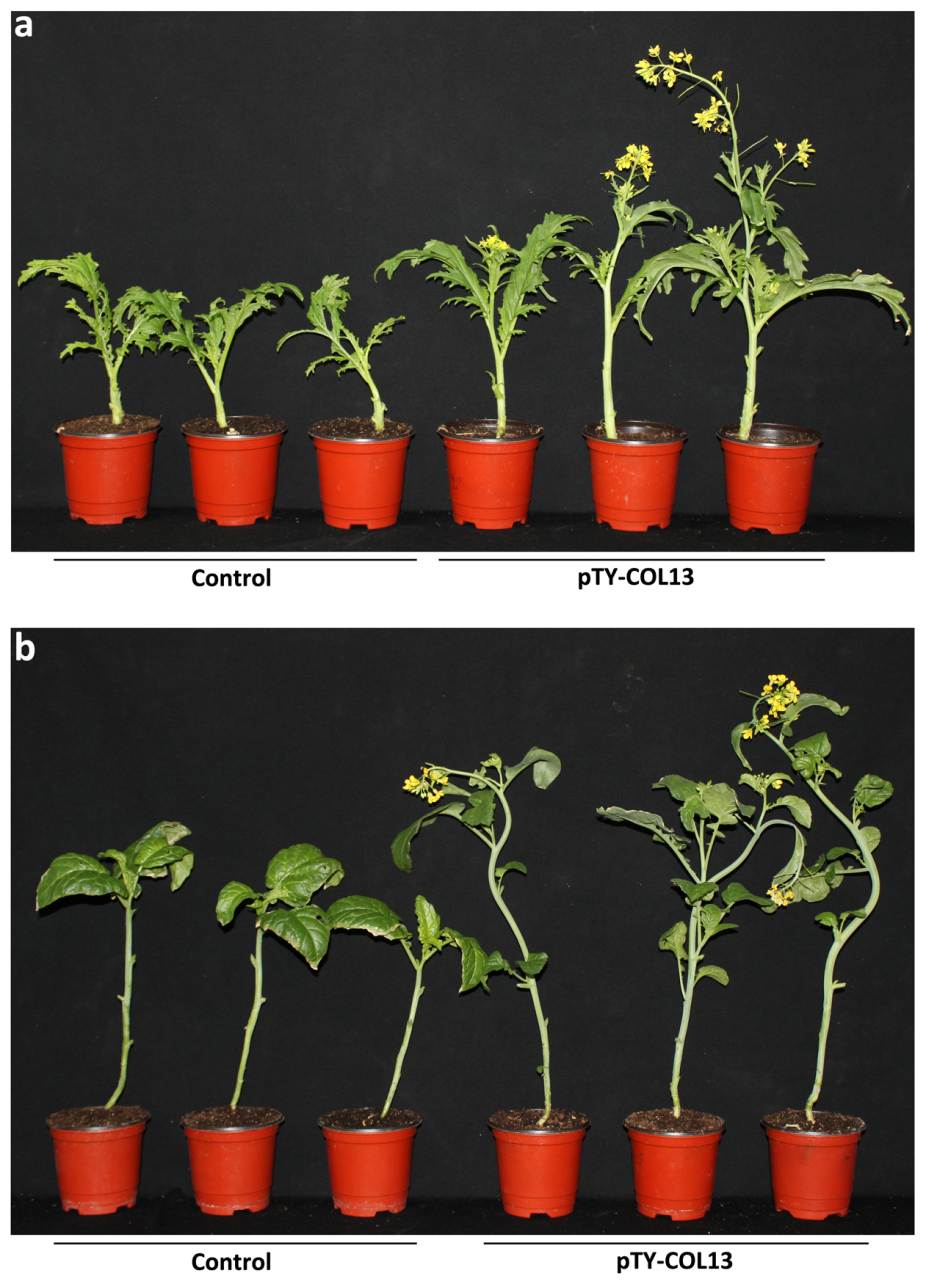


**Figure S10** Flowering phenotypes of pTY-COL13 plants (non-branching lines: **a,** T84-63; **b,** EU07) with down-regulated *COL13* expression and control plants.


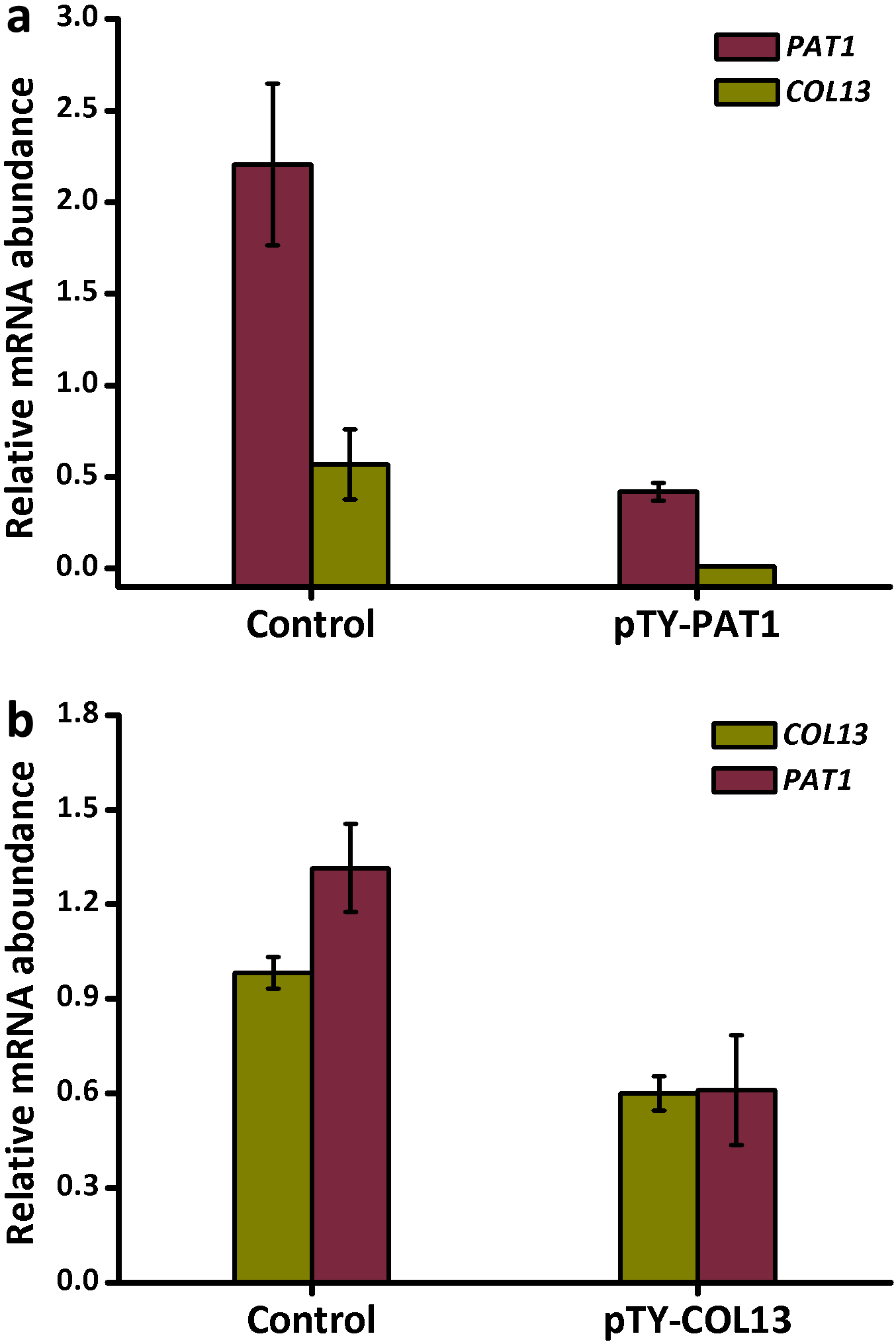


**Figure S11** Comparison of *COL13* and *PAT1* expression patterns in control, pTY-PAT1, and pTY-COL13 plants.


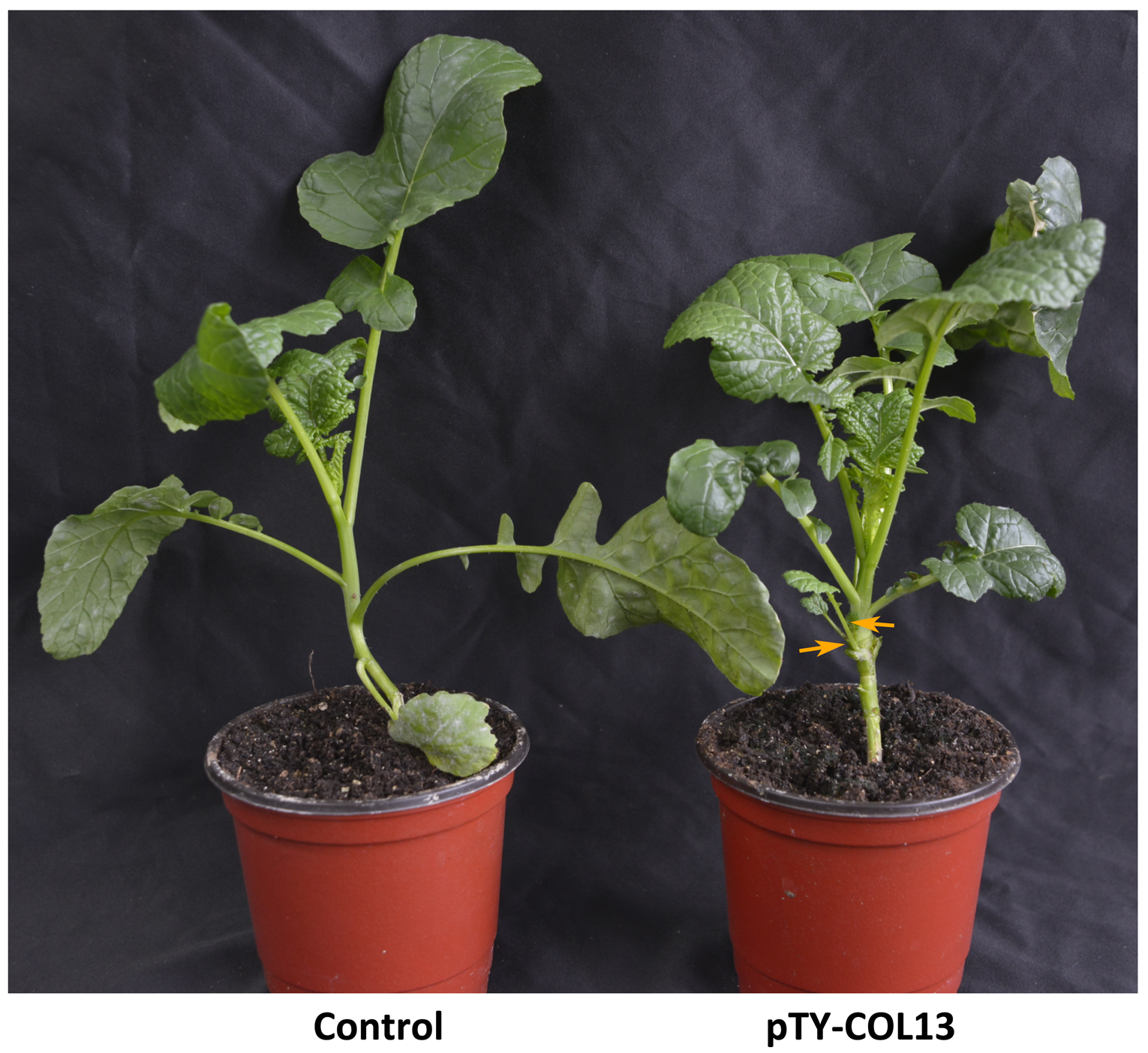


**Figure S12** Branching phenotype of pTY-COL13 plants (non-branching line: EU07) with down-regulated *COL13* expression and control plants.
